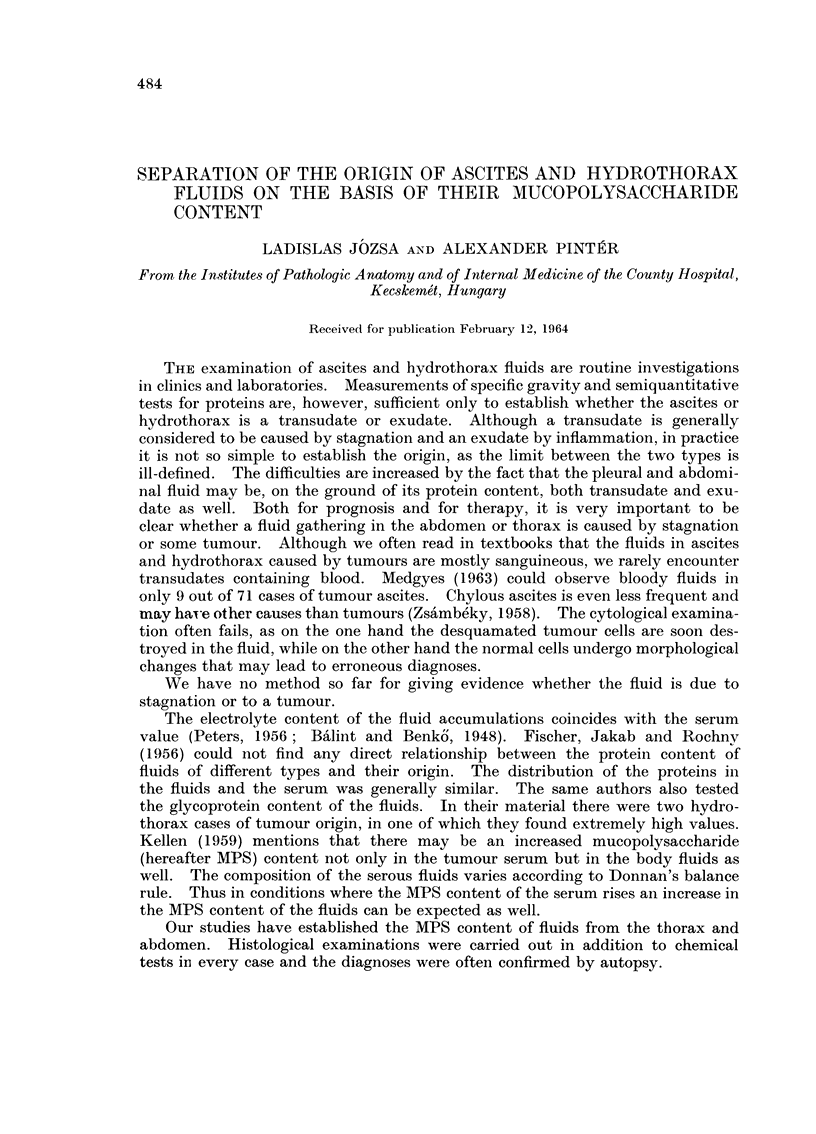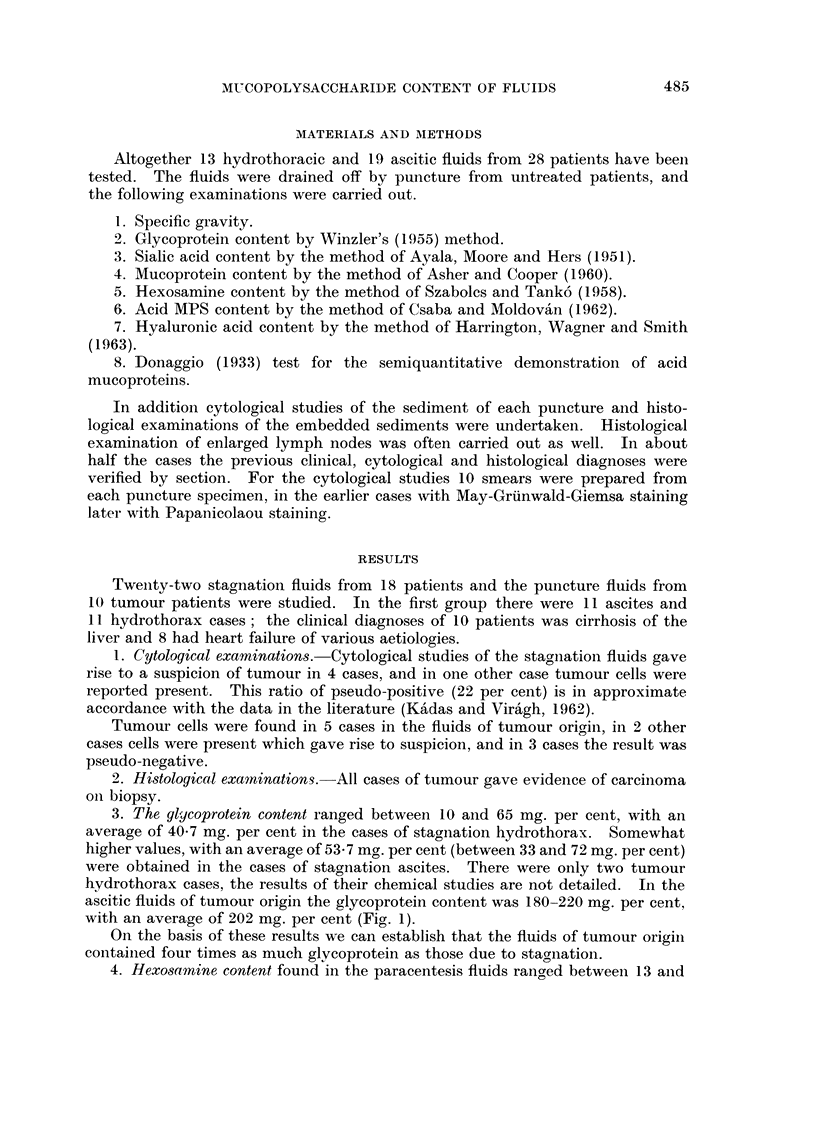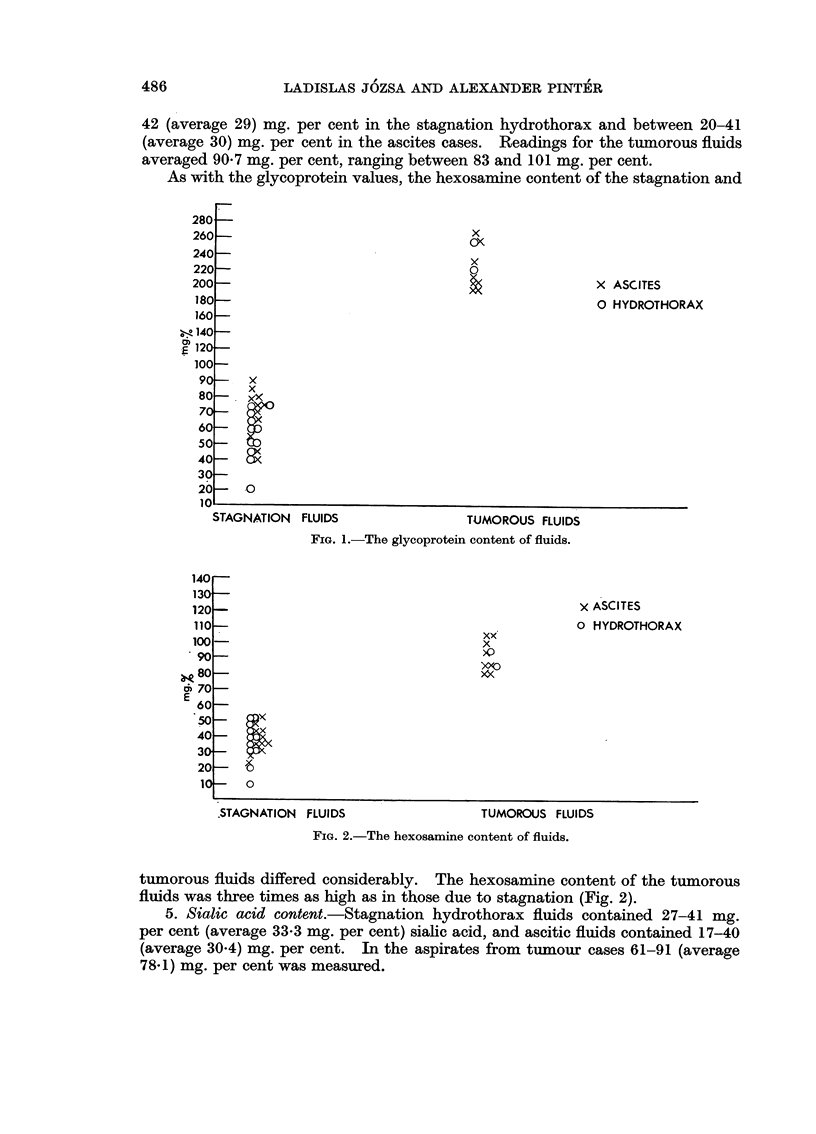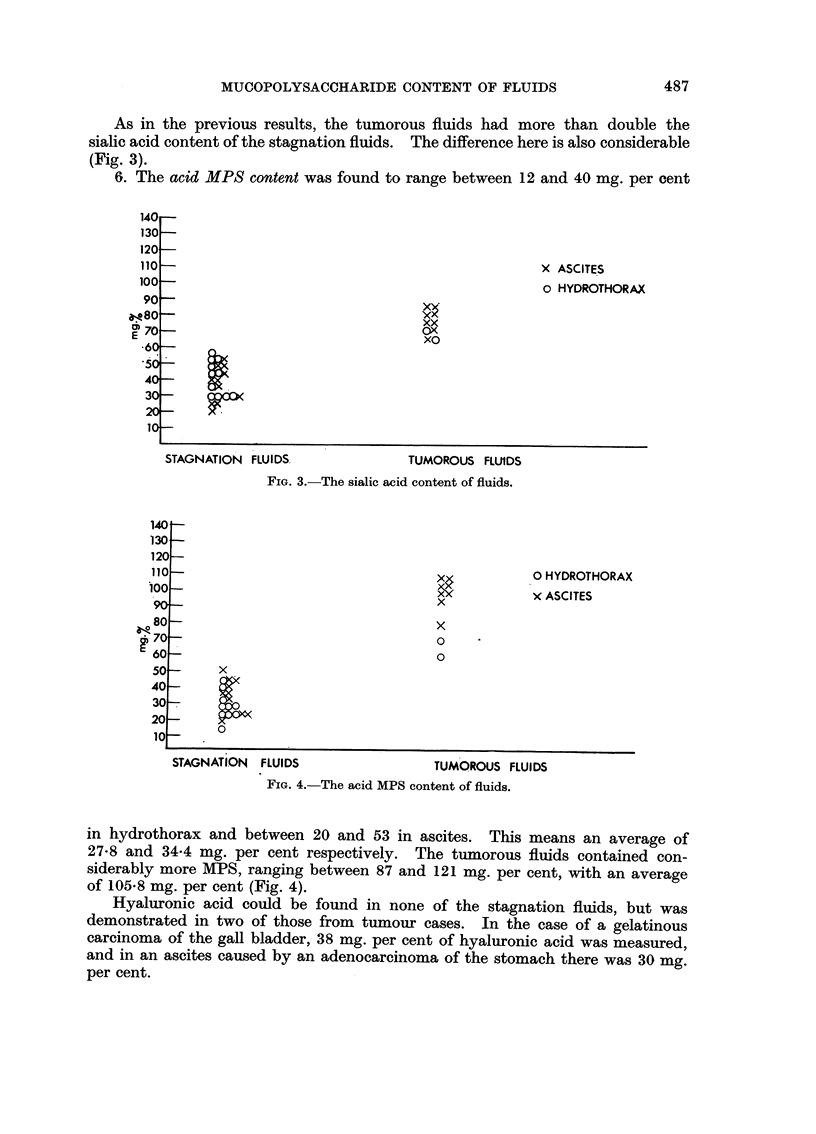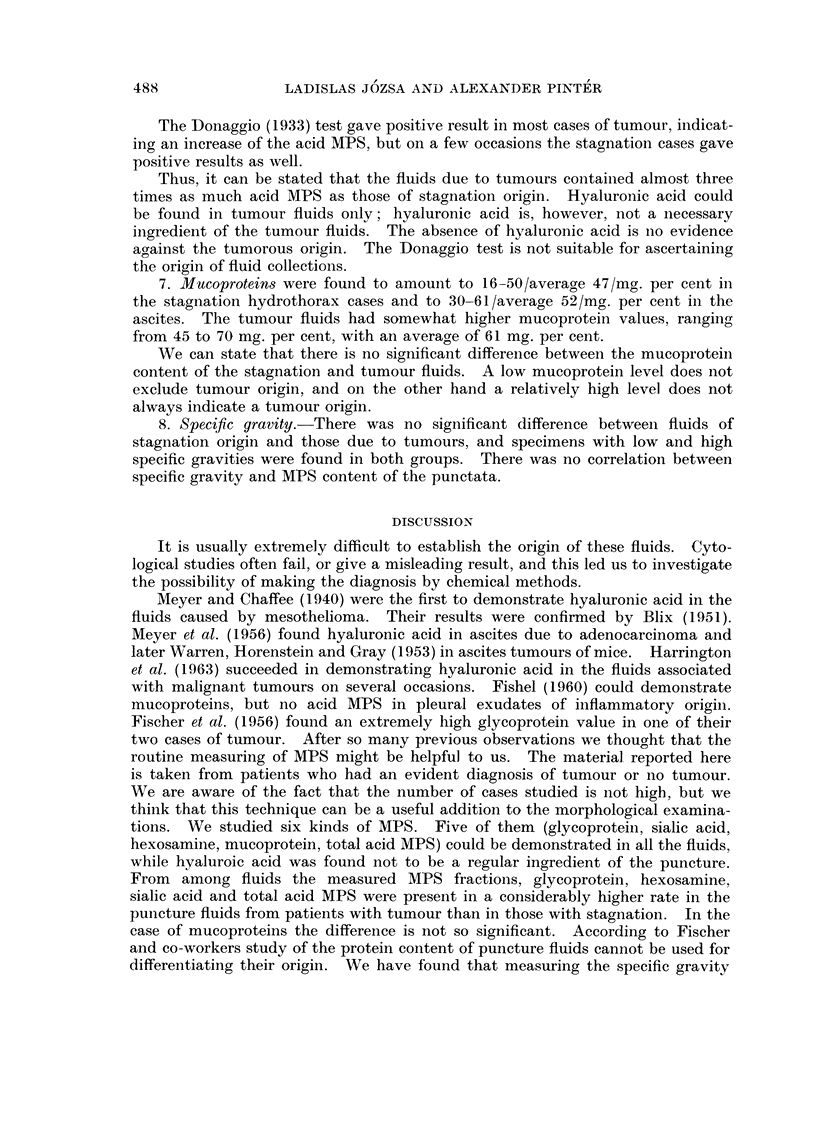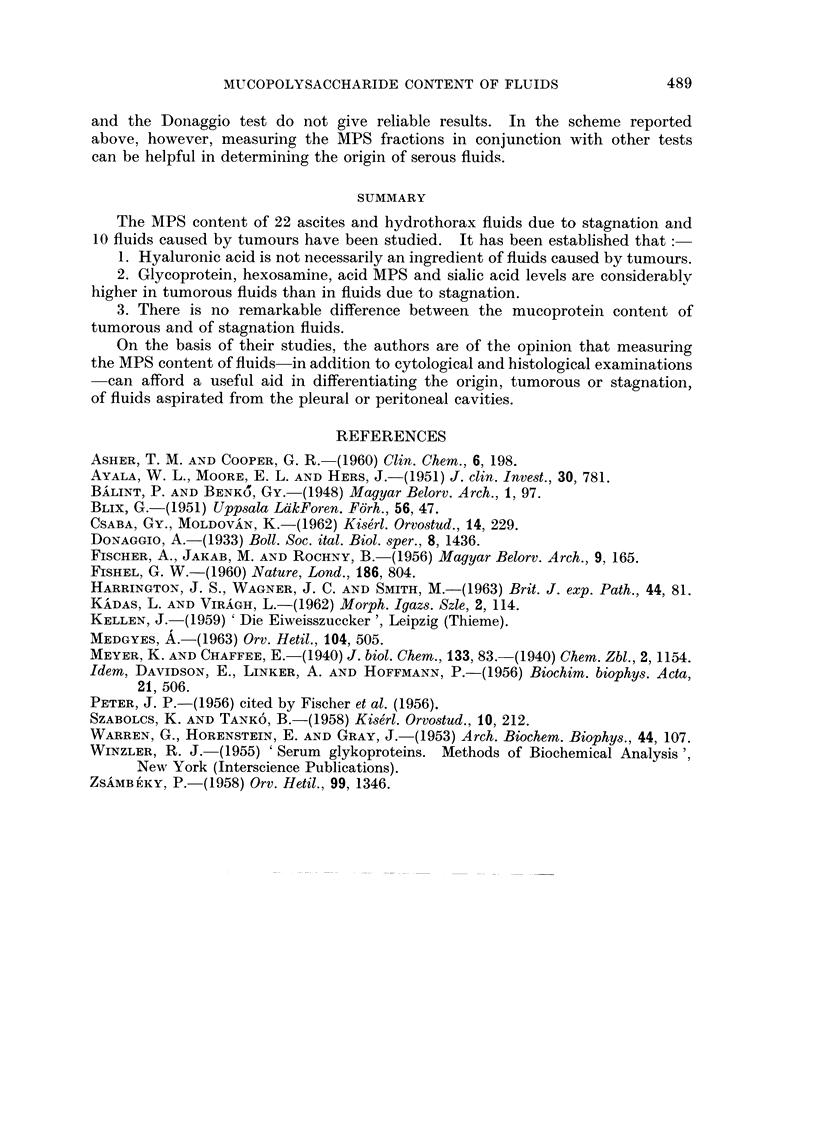# Separation of the Origin of Ascites and Hydrothorax Fluids on the Basis of their Mucopolysaccharide Content

**DOI:** 10.1038/bjc.1964.54

**Published:** 1964-09

**Authors:** Ladislas Jόzsa, Alexander Pintέr


### SEPARATION OF THE ORIGIN OF ASCITES AND HYDROTHORAX FLUIDS ON THE BASIS OF THEIR MUCOPOLYSACCHARIDE CONTENT

LADISLAS JÓZSA AND ALEXANDER PINTÉR

*From the Institutes of Pathologic Anatomy and of Internal Medicine of the County Hospital, Kecskemét, Hungary*

Received for publication February 12, 1964

THE examination of ascites and hydrothorax fluids are routine investigations in clinics and laboratories. Measurements of specific gravity and semiquantitative tests for proteins are, however, sufficient only to establish whether the ascites or hydrothorax is a transudate or exudate. Although a transudate is generally considered to be caused by stagnation and an exudate by inflammation, in practice it is not so simple to establish the origin, as the limit between the two types is ill-defined. The difficulties are increased by the fact that the pleural and abdominal fluid may be, on the ground of its protein content, both transudate and exudate as well. Both for prognosis and for therapy, it is very important to be clear whether a fluid gathering in the abdomen or thorax is caused by stagnation or some tumour. Although we often read in textbooks that the fluids in ascites and hydrothorax caused by tumours are mostly sanguineous, we rarely encounter transudates containing blood. Medgyes (1963) could observe bloody fluids in only 9 out of 71 cases of tumour ascites. Chylous ascites is even less frequent and may have other causes than tumours (Zsámbéky, 1958). The cytological examination often fails, as on the one hand the desquamated tumour cells are soon destroyed in the fluid, while on the other hand the normal cells undergo morphological changes that may lead to erroneous diagnoses.

We have no method so far for giving evidence whether the fluid is due to stagnation or to a tumour.

The electrolyte content of the fluid accumulations coincides with the serum value (Peters, 1956; Bálint and Benkő, 1948). Fischer, Jakab and Rochny (1956) could not find any direct relationship between the protein content of fluids of different types and their origin. The distribution of the proteins in the fluids and the serum was generally similar. The same authors also tested the glycoprotein content of the fluids. In their material there were two hydrothorax cases of tumour origin, in one of which they found extremely high values. Kellen (1959) mentions that there may be an increased mucopolysaccharide (hereafter MPS) content not only in the tumour serum but in the body fluids as well. The composition of the serous fluids varies according to Donnan's balance rule. Thus in conditions where the MPS content of the serum rises an increase in the MPS content of the fluids can be expected as well.

Our studies have established the MPS content of fluids from the thorax and abdomen. Histological examinations were carried out in addition to chemical tests in every case and the diagnoses were often confirmed by autopsy.

### MATERIALS AND METHODS

Altogether 13 hydrothoracic and 19 ascitic fluids from 28 patients have been tested. The fluids were drained off by puncture from untreated patients, and the following examinations were carried out.

1. Specific gravity.
2. Glycoprotein content by Winzler's (1955) method.
3. Sialic acid content by the method of Ayala, Moore and Hers (1951).
4. Mucoprotein content by the method of Asher and Cooper (1960).
5. Hexosamine content by the method of Szabolcs and Tankó (1958).
6. Acid MPS content by the method of Csaba and Moldován (1962).
7. Hyaluronic acid content by the method of Harrington, Wagner and Smith (1963).
8. Donaggio (1933) test for the semiquantitative demonstration of acid mucoproteins.

In addition cytological studies of the sediment of each puncture and histological examinations of the embedded sediments were undertaken. Histological examination of enlarged lymph nodes was often carried out as well. In about half the cases the previous clinical, cytological and histological diagnoses were verified by section. For the cytological studies 10 smears were prepared from each puncture specimen, in the earlier cases with May-Grünwald-Giemsa staining later with Papanicolaou staining.

### RESULTS

Twenty-two stagnation fluids from 18 patients and the puncture fluids from 10 tumour patients were studied. In the first group there were 11 ascites and 11 hydrothorax cases ; the clinical diagnoses of 10 patients was cirrhosis of the liver and 8 had heart failure of various aetiologies.

1. *Cytological examinations.*—Cytological studies of the stagnation fluids gave rise to a suspicion of tumour in 4 cases, and in one other case tumour cells were reported present. This ratio of pseudo-positive (22 per cent) is in approximate accordance with the data in the literature (Kádas and Virágh, 1962).

Tumour cells were found in 5 cases in the fluids of tumour origin, in 2 other cases cells were present which gave rise to suspicion, and in 3 cases the result was pseudo-negative.

2. *Histological examinations.*—All cases of tumour gave evidence of carcinoma on biopsy.

3. *The glycoprotein content* ranged between 10 and 65 mg. per cent, with an average of 40·7 mg. per cent in the cases of stagnation hydrothorax. Somewhat higher values, with an average of 53·7 mg. per cent (between 33 and 72 mg. per cent) were obtained in the cases of stagnation ascites. There were only two tumour hydrothorax cases, the results of their chemical studies are not detailed. In the ascitic fluids of tumour origin the glycoprotein content was 180–220 mg. per cent, with an average of 202 mg. per cent (Fig. 1).

On the basis of these results we can establish that the fluids of tumour origin contained four times as much glycoprotein as those due to stagnation.

4. *Hexosamine content* found in the paracentesis fluids ranged between 13 and

42 (average 29) mg. per cent in the stagnation hydrothorax and between 20–41 (average 30) mg. per cent in the ascites cases. Readings for the tumorous fluids averaged 90·7 mg. per cent, ranging between 83 and 101 mg. per cent.

As with the glycoprotein values, the hexosamine content of the stagnation and

Fig. 1.—The glycoprotein content of fluids.

Fig. 2.—The hexosamine content of fluids.

tumorous fluids differed considerably. The hexosamine content of the tumorous fluids was three times as high as in those due to stagnation (Fig. 2).

5. *Sialic acid content*.—Stagnation hydrothorax fluids contained 27–41 mg. per cent (average 33·3 mg. per cent) sialic acid, and ascitic fluids contained 17–40 (average 30·4) mg. per cent. In the aspirates from tumour cases 61–91 (average 78·1) mg. per cent was measured.

As in the previous results, the tumorous fluids had more than double the sialic acid content of the stagnation fluids. The difference here is also considerable (Fig. 3).

6. The *acid MPS content* was found to range between 12 and 40 mg. per cent

Fig. 3.—The sialic acid content of fluids.

Fig. 4.—The acid MPS content of fluids.

in hydrothorax and between 20 and 53 in ascites. This means an average of 27·8 and 34·4 mg. per cent respectively. The tumorous fluids contained considerably more MPS, ranging between 87 and 121 mg. per cent, with an average of 105·8 mg. per cent (Fig. 4).

Hyaluronic acid could be found in none of the stagnation fluids, but was demonstrated in two of those from tumour cases. In the case of a gelatinous carcinoma of the gall bladder, 38 mg. per cent of hyaluronic acid was measured, and in an ascites caused by an adenocarcinoma of the stomach there was 30 mg. per cent.

The Donaggio (1933) test gave positive result in most cases of tumour, indicating an increase of the acid MPS, but on a few occasions the stagnation cases gave positive results as well.

Thus, it can be stated that the fluids due to tumours contained almost three times as much acid MPS as those of stagnation origin. Hyaluronic acid could be found in tumour fluids only; hyaluronic acid is, however, not a necessary ingredient of the tumour fluids. The absence of hyaluronic acid is no evidence against the tumorous origin. The Donaggio test is not suitable for ascertaining the origin of fluid collections.

7. *Mucoproteins* were found to amount to 16-50/average 47/mg. per cent in the stagnation hydrothorax cases and to 30–61/average 52/mg. per cent in the ascites. The tumour fluids had somewhat higher mucoprotein values, ranging from 45 to 70 mg. per cent, with an average of 61 mg. per cent.

We can state that there is no significant difference between the mucoprotein content of the stagnation and tumour fluids. A low mucoprotein level does not exclude tumour origin, and on the other hand a relatively high level does not always indicate a tumour origin.

8. *Specific gravity.*—There was no significant difference between fluids of stagnation origin and those due to tumours, and specimens with low and high specific gravities were found in both groups. There was no correlation between specific gravity and MPS content of the punctata.

### DISCUSSION

It is usually extremely difficult to establish the origin of these fluids. Cytological studies often fail, or give a misleading result, and this led us to investigate the possibility of making the diagnosis by chemical methods.

Meyer and Chaffee (1940) were the first to demonstrate hyaluronic acid in the fluids caused by mesothelioma. Their results were confirmed by Blix (1951). Meyer *et al.* (1956) found hyaluronic acid in ascites due to adenocarcinoma and later Warren, Horenstein and Gray (1953) in ascites tumours of mice. Harrington *et al.* (1963) succeeded in demonstrating hyaluronic acid in the fluids associated with malignant tumours on several occasions. Fishel (1960) could demonstrate mucoproteins, but no acid MPS in pleural exudates of inflammatory origin. Fischer *et al.* (1956) found an extremely high glycoprotein value in one of their two cases of tumour. After so many previous observations we thought that the routine measuring of MPS might be helpful to us. The material reported here is taken from patients who had an evident diagnosis of tumour or no tumour. We are aware of the fact that the number of cases studied is not high, but we think that this technique can be a useful addition to the morphological examinations. We studied six kinds of MPS. Five of them (glycoprotein, sialic acid, hexosamine, mucoprotein, total acid MPS) could be demonstrated in all the fluids, while hyaluroic acid was found not to be a regular ingredient of the puncture. From among fluids the measured MPS fractions, glycoprotein, hexosamine, sialic acid and total acid MPS were present in a considerably higher rate in the puncture fluids from patients with tumour than in those with stagnation. In the case of mucoproteins the difference is not so significant. According to Fischer and co-workers study of the protein content of puncture fluids cannot be used for differentiating their origin. We have found that measuring the specific gravity and the Donaggio test do not give reliable results. In the scheme reported above, however, measuring the MPS fractions in conjunction with other tests can be helpful in determining the origin of serous fluids.

### SUMMARY

The MPS content of 22 ascites and hydrothorax fluids due to stagnation and 10 fluids caused by tumours have been studied. It has been established that :—

1. Hyaluronic acid is not necessarily an ingredient of fluids caused by tumours.
2. Glycoprotein, hexosamine, acid MPS and sialic acid levels are considerably higher in tumorous fluids than in fluids due to stagnation.
3. There is no remarkable difference between the mucoprotein content of tumorous and of stagnation fluids.

On the basis of their studies, the authors are of the opinion that measuring the MPS content of fluids—in addition to cytological and histological examinations—can afford a useful aid in differentiating the origin, tumorous or stagnation, of fluids aspirated from the pleural or peritoneal cavities.

### REFERENCES

Asher, T. M. and Cooper, G. R.—(1960) *Clin. Chem.*, **6**, 198.
Ayala, W. L., Moore, E. L. and Hers, J.—(1951) *J. clin. Invest.*, **30**, 781.
Bálint, P. and Benkő, Gy.—(1948) *Magyar Belorv. Arch.*, **1**, 97.
Blix, G.—(1951) *Uppsala LäkForen. Förh.*, **56**, 47.
Csaba, Gy., Moldován, K.—(1962) *Kisérl. Orvostud.*, **14**, 229.
Donaggio, A.—(1933) *Boll. Soc. ital. Biol. sper.*, **8**, 1436.
Fischer, A., Jakab, M. and Rochny, B.—(1956) *Magyar Belorv. Arch.*, **9**, 165.
Fishel, G. W.—(1960) *Nature, Lond.*, **186**, 804.
Harrington, J. S., Wagner, J. C. and Smith, M.—(1963) *Brit. J. exp. Path.*, **44**, 81.
Kádas, L. and Virágh, L.—(1962) *Morph. Igazs. Szle*, **2**, 114.
Kellen, J.—(1959) ' Die Eiweisszuccker ', Leipzig (Thieme).
Medgyes, Á.—(1963) *Orv. Hetil.*, **104**, 505.
Meyer, K. and Chaffee, E.—(1940) *J. biol. Chem.*, **133**, 83.—(1940) *Chem. Zbl.*, **2**, 1154.
*Idem*, Davidson, E., Linker, A. and Hoffmann, P.—(1956) *Biochim. biophys. Acta*, **21**, 506.
Peter, J. P.—(1956) cited by Fischer *et al.* (1956).
Szabolcs, K. and Tankó, B.—(1958) *Kisérl. Orvostud.*, **10**, 212.
Warren, G., Horenstein, E. and Gray, J.—(1953) *Arch. Biochem. Biophys.*, **44**, 107.
Winzler, R. J.—(1955) ' Serum glykoproteins. Methods of Biochemical Analysis ', New York (Interscience Publications).
Zsámbéky, P.—(1958) *Orv. Hetil.*, **99**, 1346.